# Recovery After Genocide: Understanding the Dimensions of Recovery Capital Among Incarcerated Genocide Perpetrators in Rwanda

**DOI:** 10.3389/fpsyg.2019.00637

**Published:** 2019-04-16

**Authors:** Kevin Barnes-Ceeney, Lior Gideon, Laurie Leitch, Kento Yasuhara

**Affiliations:** ^1^Henry C. Lee College of Criminal Justice and Forensic Sciences, University of New Haven, West Haven, CT, United States; ^2^John Jay College of Criminal Justice, New York, NY, United States; ^3^Threshold GlobalWorks, LLC, New York, NY, United States

**Keywords:** genocide, rwanda, trauma, posttraumatic stress, recovery capital

## Abstract

Utilizing survey data from 302 men and women incarcerated in the Rwandan correctional system for the crime of genocide, and structured interviews with 75 prisoners, this mixed methods study draws on the concept of recovery capital to understand how individuals convicted of genocide navigate post-genocide healing. Genocide smashes physical and human capital and perverts social and cultural capital. Experiencing high levels of posttraumatic stress symptoms with more than two-thirds of the sample scoring above typical civilian cut-off levels, raised levels of depression, and high levels of anxiety, and failing physical health, the genocide perpetrators require multiple sources of recovery capital to foster internal resilience as they look forward to rebuilding their own lives.

## Introduction

Post-genocide Rwanda has experienced a concerted, multi-dimensional program of unity and reconciliation aimed at bringing peace and prosperity to the country. Utilizing survey data from 302 men and women incarcerated in the Rwandan correctional system for the crime of genocide, and structured interviews with 75 prisoners, this mixed methods study draws on the concept of recovery capital to understand how individuals convicted of genocide experience and adapt to stress, distress, and trauma. Applying Maslow's hierarchy of needs to recovery capital, this paper explores the interconnections between social, cultural, physical and human capital and symptoms of posttraumatic stress after genocide. Maslow's hierarchy of needs provides an important framework for examining the “building blocks” of recovery capital that may contribute to successful post-genocide healing for perpetrators.

## Literature Review

### The Trauma of Genocide

State-sanctioned incitement to hate fueled the 1994 Rwandan genocide, where in just 100 days between 800,000 and 1,000,000 Tutsi and moderate Hutu were massacred with guns, machetes, and nail studded clubs (Reyntjens, 2004). It is estimated that up to one million individuals participated in horrific genocidal acts of killing and looting (Waldorf, 2006). Referred to as the most efficient genocide in modern times (Kuperman, 2004) owing to the speed and scale of the killing, and the “intimate genocide” (Staub and Pearlman, 2001) due to the close nature of the killing, the Rwandan genocide involved many, was swift and was particularly brutal. Atrocities included impaling male victims from anus to mouth, female breast oblation (Taylor, 1999), using HIV through rape as a biological weapon (Baines, 2003), and smashing babies against walls (Rutazibwa and Rutayisire, 2007). Scholars have noted that many genocide perpetrators were placed under “extreme pressure” (Schaal et al., 2012, p. 450) to commit genocidal atrocities. A confluence of pressures led to and sustained the genocide. Key precipitating factors included pervasive narratives of a Tutsi threat to the Rwandan social body (McDoom, 2012), pseudo-ethno categories promulgated by Belgian colonists under the guise of the Hamitic Hypothesis (Taylor, 1999; Eltringham, 2006), a frustration of basic human needs through poverty (Staub, 2003), and fear of personal violence if an individual refused to participate (Smeulers and Hoex, 2010).

The trauma engendered by the 1994 Rwandan genocide directly impacts victims, eyewitnesses, genocide perpetrators, and those immediately entering Rwanda post-genocide to engage in nation reconstruction. Therefore, we can understand the category of genocide “survivors” as comprising victims, eyewitnesses, perpetrators, and post-conflict reconstructors. Genocide perpetrators may experience symptoms of posttraumatic stress by witnessing their own actions, and the actions of other perpetrators (Schaal et al., 2012). Certainly, DSM-IV and 5 acknowledges that both being forced to commit violence and witnessing violent acts can generate posttraumatic stress (American Psychiatric Association, 2013).

A small number of studies have examined the prevalence of traumatic stress among the Rwandan population post-genocide. (Fodor et al., 2015) examining 465 genocide survivors found raised PTSD scores a mean PCL-C score of 31.4 points (*SD* = 15.8, max. 79). (Rugema et al., 2015), drawing on a random population sample of 913 Rwandans estimated that 13.6% were suffering from PTSD. One study has previously examined levels of PTSD symptoms among Rwandan genocide perpetrators. Schaal et al. (2012) studying 269 genocide perpetrators found 13.5% met diagnostic criteria for PTSD. Schaal et al. (2012) found that genocide perpetrators were continually reliving their genocidal actions and desperately seeking redemption. It is within this context that the current study aims to examine how key components of recovery capital—social capital, cultural capital, physical capital, and human capital—interact with and impact post-genocide healing.

### The Concept of Recovery Capital

Emerging from the addictions field, the concept of recovery capital encompasses the sum of resources that may facilitate the lived experience of recovery (Granfield and Cloud, 1999, 2001; Cloud and Granfield, 2008; Laudet and White, 2008; Best and Laudet, 2010). Cloud and Granfield (2008) note that although substance misuse occurs within all levels of society it is differentially experienced, such that recovery from substance misuse varies widely. They observe that successful recovery depends upon environmental contexts, personal characteristics of the user, and the availability of particular resources. Cloud and Granfield conclude that recovery capital comprises four key components: social capital, cultural capital, physical capital, and human capital. Individuals who are “resource-rich” in each component have a greater likelihood of exiting from substance misusing situations. Furthermore, the various capitals are potentially convertible, for example social capital may be converted into human capital, and cultural capital may be converted into economic capital, although such conversions often take time and economic resources (Bourdieu, 1986).

### Social Capital

Social capital “exist in the relations among persons” (Coleman, 1988, pp. S100-101). Drawing upon Granovetter's (1985) notion of “embeddedness,” Coleman emphasizes the importance of nurturing relational trust, having clarity around expectations, and thereby developing accepted shared norms. Such social capital may be conceptualized as horizontal ties and relationships among relatively homogenous groups, or vertical ties that connect levels between hierarchical levels (Baum and Ziersch, 2003).

Bonding social capital as a form of “social capitals” (Whitley and McKenzie, 2005, p. 73), is a function of collective actions between relatively homogenous groups (Putnam, 2000; Lo, 2010). Such dense ties are typically found in relationships between the individual and his or her family, religious institution, and immediate community (Coleman, 1988). Bonding social capital tends to be inward-looking, providing individual and group ontological security, often through the process of distancing others (Young, 1999). Bridging social capital functions between the individual and distant friends and associates (Lo, 2010), and community groups and movements (Putnam, 2000). Loose bridging ties may be formed through the generation of transitive trust (Fountain, 1998), whereby trust afforded to a closer tie is automatically extended to those with looser ties because of the strength of the original bonding social capital.

Vertical ties are a function of “linking social capital” (Szreter and Woolcock, 2004) which provides vertical connections between the individual and those with greater power (Granovetter, 1973). Such vertical ties, as Granovetter (1973) suggests, can lead to potential mobility opportunities, by providing access to power, wealth and social status to individuals and groups (Woolcock, 1998).

Critical to the development of bonds, bridges and links between individuals and groups is the development of mutual trust and solidarity. The development of shared norms promotes community solidarity in a cyclical trust-reinforcing process. Mutual trust lies at the heart of “collective efficacy” a group-level attribute defined as “a group's shared belief in its conjoint capabilities to organize and execute the courses of action required to produce given levels of attainments” (Bandura, 1997, p. 477). Acknowledging the importance of collective efficacy—a community's perception that they are able to intervene for the common good—emphasizes the agentic aspects of social capital, and generates community “expectations for action” (Sampson, 2003, S. 59).

Scholars have noted that high levels of social capital speed individual and community recovery after natural and man-made disasters such as hurricanes in the Caribbean region (Adger et al., 2005, the Asian financial crisis (Wetterberg, 2005), the Tsunami in Sri Lanka (Minamoto, 2010), and the Wenchuan earthquake in Sichuan, China (Huang and Wong, 2013). Wider and denser social networks can facilitate recovery by expediting access to resources and guidance, engendering community mobilization, and raising the cost of community exit (Landau and Saul, 2004; Aldrich, 2011). In relation to Rwandan post-genocide recovery, the need to promote social capital has been recognized (Scholte and Ager, 2014; Verduin et al., 2014; Mahr and Campbell, 2016). The violence of mass conflict and genocide destroys social ties, extirpates mutual trust, and shatters social cohesion. Paradoxically, mass conflict and genocide has the potential to strengthen and concretize horizontal and vertical ties. Colletta and Cullen (2000) provide a nuanced understanding of the role of social capital both during and after the Rwandan genocide. For participating Hutu, Colletta and Cullen suggest, the genocide was “a powerful communal-building experience” (p. 41) where bonding ties were amplified. For Tutsi's and moderate-Hutu's bonding ties “founded on fear and survival” (p. 42) were similarly strengthened. Simultaneously, genocide destroys previously nurturing ties, through the loss of family members and neighbors, and the spread of uncertainty and mistrust (Zuckerman and Greenberg, 2004). Such a “perversion of social capital” (p. 40) renders post-genocide recovery a gargantuan task.

In post-genocide Rwanda, efforts to build social capital emanate largely from the National Unity Reconciliation Commission (NURC). Laws criminalizing ethnic divisionism and genocide revisionism (Thomson, 2011), and policies promoting “Rwandan-ness” (Vandeginste, 2014), seek to foster solidarity and trust among the Rwandan population. Although the nurturing of solidarity and trust is critical for the development of sustainable bridging ties, whether all Rwandans accept such forced unity and reconciliation remains moot (Thomson, 2011). Specific projects, such as the *Association Modeste et Innocent*, a non-profit organization that brings genocide perpetrators and survivors together through a process of intensive counseling and support (Dominus and Hugo, 2014) provide considerable opportunities to nurture bonding social capital between individuals, reinforcing notions of Rwandan-ness. Coupled with the acceptance of “unifying” laws and policies, and a strong individual desire for a peaceful future, there may be an iterative conversion of such social capitals to cultural capital.

### Cultural Capital

Drawing on the work of Bourdieu (1973), cultural capital arises from and is shaped by historical, social, and economic processes as they impact families and communities (Weine et al., 2004). Cultural capital comprises of three distinct forms: the embodied state, the objectified state and the institutionalized state (Bourdieu, 1986). Capital, in the form of acceptance of and adherence to cultural norms becomes embodied when it becomes an integral part of the individual. Embodied capital is implicitly transmitted in family and institutional settings. The unconscious inheritance of cultural wealth is similar to Cohen's (1955) observation that middle class boys are advantaged in the school setting because they have already internalized middle class values. Cloud and Granfield (2008) suggest that the internalization of the norms of the dominant culture help the development of “stakes in conformity” (Toby, 1957), which can assist the substance misuse recovery process. Objectified cultural capital refers to objects such as paintings writings, and artifacts which may be appropriated through ownership or consumption. Thus, objectified cultural capital functions both materially and symbolically to endow the receiver with perceived cultural wealth. Finally, institutionalized cultural capital exists in form of qualifications and status endowed through institutional recognition. Bourdieu (1986) notes that such institutionalized cultural capital enriches the individual through its scarcity.

Just as there is a “perversion of social capital” (Colletta and Cullen, 2000, p. 40) during genocide, cultural capital becomes similarly exaggerated, contorted and misused. Embodied capital takes the form of hostility, resentment, and fear toward groups defined as “other.” Narratives of othering are often shaped by perceived and actual historical wrongs (Noor et al., 2008) that become integral to current individual and group identity. The other becomes perceived as a critical threat to the social body requiring classification, containment, and eventually extermination.

Perverted cultural capital becomes objectified through the generation and use of ethnic identification papers and identifying symbols such as yellow stars or blue-checked scarfs (Stanton, 2004, 2013). Physical characteristics such as slight differences in nose and lip shape or variations in skin tone are an inherited form of objectified cultural capital. Such physical markers of status, power, and difference cannot be shed. Markers of status become institutionalized through government policies that exclude and polarize. Specifically, Rwanda experienced a perversion of institutionalized cultural capital through the creation and distribution of lists of Tutsi and moderate Hutu's (African Rights, 1995), travel restrictions on Tutsi's (Kalimba, 2015), the creation and expansion of an all-Hutu government army (Stanton, 2004), the mass importation of weapons (Alusala, 2004), and the government-backed spread of genocide ideology through radio and newspaper propaganda. Certainly, the focus of mass torture and killing was on “ridding the polity of a categorical enemy” (Taylor, 1999, p. 140). Sustainable post-genocide recovery requires the reorganization and realignment of embodied, objectified, and institutionalized cultural capital, to engender peaceful and harmonious co-existence between previously fractured groups.

Efforts to build post-genocide cultural capital include the governmental promulgation of an historical narrative that pre-colonial Rwanda enjoyed unity and harmony (Gready, 2010), and assertions that, in relation to the 1994 Rwandan genocide, there is just “one truth” (Human Rights Watch, 2008). The “truth” of the Rwandan genocide includes three critical tenets. Firstly, the Belgian colonial administration were assisted by the Catholic Church to introduce divisive ethnic categories in Rwanda and so bear considerable responsibility for the subsequent genocidal crimes. Secondly, the genocide was organized by Hutu political leaders who misled the Hutu population to participate in the genocide. Finally, although there is some recognition that soldiers in the Rwandan Patriotic Army killed Hutu civilians during the genocide, these killings were unfortunate acts of war or revenge. Such “truth” seeks to concretize victim—perpetrator dichotomies, thereby strengthening embodied and objectified cultural capital. Overtime, there may be a conversion of this cultural capital into bonding social capital, although the unifying message is somewhat contradictory.

### Physical Capital

The third key component of recovery capital is physical capital. Physical capital is “wholly tangible, being embodied in observable material form” (Coleman, 1990, p. 304). Related to the capacity to generate further wealth, physical capital is “non-human assets” (Qiang et al., 2004), and includes possession of components critical to the means of production such as plant, machines, buildings (Coleman, 1990), tools (Throsby, 1999), and equipment (Harkness, 1978). Viewing physical capital as a financial capital safety net, Cloud and Granfield (2008) suggest that it includes “income, savings, property, investments, and other tangible assets that can be converted to money” (p. 1973). They suggest that financial capital may help substance users' access help and resources, or provide temporary respite from environmental cues and triggers through leaves of absence or extended vacations.

Describing Rwanda immediately after the 1994 genocide (Gourevitch, 1995) writes that the country was “a virtually empty treasury” (para. 23), with factories and machines, power facilities sabotaged, and water systems polluted with corpses. Schools, hospitals, roads, and offices lay in ruins and genocidal looters had stolen all money from banks (Clark, 2018). The economic impact of such countrywide devastation reverberated for years after the genocide. Examining the economic impact of the Rwandan genocide 10 years later, Lopez and Wodon (2005) estimate that the genocide led to a 25–30% decrease in Rwanda's per capita GDP. Genocide and mass conflict seriously deplete physical capital impeding both short and long-term recovery. Physical capital, in the form of economic capital is perhaps the easiest form of capital to convert to other forms of capital. In post-genocide Rwanda the conversion of physical capital to human capital is an important feature of the reconstruction process. It facilitates the learning of “modern” farming techniques, engendering a livelihood offering more than subsistence farming. Such excess can then potentially be converted to strengthened physical capital. Although physical capital can be converted over time to individual social and cultural capital, such conversion may undermine unity and reconciliation efforts. Rwanda has the highest wealth inequality rate in East Africa, with the income of the richest 10% being 3.2% more than the poorest 40% (Asiimwe, 2017). Unless, however, income inequality and extreme poverty is addressed, economic capital is likely to reinforce divisions within Rwandan society.

### Human Capital

The fourth, and perhaps most extensive, component of recovery capital is human capital. Cloud and Granfield (2008) suggest that human capital includes “knowledge, skills, educational credentials, health, mental health, and other acquired or inherited traits essential for optimal negotiation of daily life” (p. 1974). Becker (1994) suggests that education and training are the most important investments we make in human capital, observing that high school and college educational credentials significantly raise individual income. More recently, scholars have noted that although educational qualifications raise income, those engaged in career and technical education (CTE) programs have significantly higher earnings than those receiving credentials in non-vocational areas of study (Jacobson and Mokher, 2009; Dadgar and Weiss, 2012). Similarily, human capital theory (Becker and Tomes, 1986) posits that improvements in a parent or child's skills lead to the generation of new skills and abilities that can lead to intergenerational mobility.

Just as genocide destroys and perverts social and cultural capital, human capital is similarly ravaged. Examining the impact of the Rwandan genocide on educational outcomes, Akresh and de Walque (2008) found that there was an 18.3% drop in average educational attainment among children exposed to the genocide. Living through genocidal acts, whether as perpetrator, victim, or bystander, may lead to the development of mental health symptoms including anxiety, and depression and posttraumatic stress (Schaal et al., 2012). Scholars have also noted that experience of trauma may inhibit an individual's ability to develop future positive relationships with others (Ronel and Elisha, 2011).

Cloud and Granfield (2008) note that the three elements of human capital particularly pertinent to substance abuse recovery are heredity, mental health, and employability. They suggest that genetic mechanisms may influence physical and mental health and note the prevalence of co-occurring addictive and mental health disorders among the general population. Cloud and Granfield (*ibid*) also suggest that employment related skills are critical to addiction recovery as they can “provide a legitimate source of steady income” (p. 1974).

Bourdieu's (1986) criticism that human capital theory never frees itself from economism is pertinent and applicable here. For Cloud and Granfield (2008), human capital is an important component of recovery capital because it provides the means to develop economic and physical capital. Certainly, as Christie (2004) reminds us, production, monetary gain and consumption comprise the heart of modernity, and certainly are the drivers of human and physical capital.

Both physical and mental health are critical components of human capital essential to recovery from trauma. Subica et al. (2012) studied the relationship between trauma, depression, substance abuse, mental health, and physical health in those with severe mental illness. Their results indicate that trauma exposure and PTSD were associated with depression, substance use, as well as overall mental and physical health. Levine (1997) suggests that our ability to respond appropriately when faced with danger depends upon a number of factors, including the degree and intensity of the traumatic event, support from family and friends, age, physical health and fitness, experience of ongoing stress and fatigue, genetic resilience, learned responses to trauma, and self-efficacy in relation to trauma. Physical health status is particularly important. The residual energy generated by fight, flight, or freeze responses to traumatic events can cause a myriad of physical and mental health problems, including anxiety, depression, behavioral problems, and short and long term somatic symptoms. Traumatic stress symptoms arise from a post-trauma “tornado of energy” (Levine, 1997, p. 20) that becomes locked inside the body and mind. Such undigested trauma is stored as physiological reactivity (Scaer, 2005), often manifesting itself as loose bowels, stomach upsets, headaches and migraines, fatigue, hypertension, diabetes, and heart disease (Leitch et al., 2009).

The four key components of recovery capital: social capital, cultural capital, physical capital, and human capital discussed above, appear relevant and appropriate for considering healing and recovery from the trauma of genocide. A somewhat complementary framework for thinking about post-genocide recovery is Maslow's hierarchy of human need (Maslow, 1943, 1954). Specifically considering the Rwandan genocide Uwamaliya and Smith (2017) note the relevance of Maslow's hierarchy in thinking about recovery for genocide survivors. After genocide and mass conflict there are multiple physiological/basic needs which need to be met, such as water, sanitation, food, and shelter during post-genocide recovery. Their study further notes the difficulty in achieving access to clean water, where the goal of the Rwandan Government is to enable such access to all people by 2020. Addressing these factors in addition to higher level needs, is important for perpetrators as they are released back into the same communities as survivors.

Maslow's hierarchy of needs, which outlines specific needs that are considered necessary for individuals' to achieve a fulfilling and happy life, is one of the most prominent theories behind human behavior. Maslow's original scheme depicted five hierarchical levels, as the lower level is satisfied; a higher level of need emerges. The bottom five levels, presented in Table 1, indicate the original hierarchy of needs, in order of need, with the bottom (physiological/basic needs) needing to be met before one could move to desire and “need” the next level (safety needs). There has been broad support found for Maslow's theory and research into a hierarchy of human needs (Chulef et al., 2001). The highest level, self-transcendence, was noted by Maslow later, as a step where the individual may desire to identify with something greater than oneself. Maslow's model is a linear hierarchy that implies that one need must be met before the next need can be addressed. However, it is also possible to conceptualize needs in a more holistic way in which key categories of need interact and add to or deplete each other. Such an approach avoids overly-simplistic linear cause and effect intellections and instead acknowledges the fluidity and reflexivity of recovery and resilience.

**Table 1:** Maslow's hierarchy of needs.

**Motivational level**	**Definition**
Self-transcendence	“Highest and most inclusive or holistic levels of human consciousness, behaving and relating, as ends rather than means, to oneself, to significant others, to human beings in general, to other species, to nature, and to the cosmos.” (Maslow, 1993, p. 269)
Self-actualization	Concern with realization and fulfillment of one's potential
Esteem needs	Concern with recognition, status, and respect for others
Social belonging	Concern with acceptance into a group
Safety needs	Concern with Physical, economical, and personal security
Physiological / basic need	Concern with maintenance of life

*Edited from Koltko-Rivera (2006)*.

Given the relative youth of the concept of recovery capital, it is useful to consider it in relation to the more establish hierarchy of need. The concept of recovery capital can be viewed utilizing Maslow's broad categories of needs, regardless of whether one subscribes to the conceptual model of a linear hierarchy. Dimensions of human capital and physical capital correspond with physiological and safety needs. Social and cultural capital straddle social belonging and esteem needs. The educational attainment component of human capital also seems to correspond with esteem needs when educational qualifications afford individuals status and respect.

Studies have examined the validity and practical use of Maslow's theory within different cultural contexts. Gambrel and Cianci (2003) viewed, through a management lens, the application of Maslow's hierarchy within a collectivistic culture. The authors found that although the needs would be similar, the hierarchical order of the needs may differ. When looking specifically at China, they found that a sense of social belonging would be the most basic need, arguing that becoming accepted into society is the only way to satisfy physiological / basic survival need as well as safety needs. Potentially, this is indicative of the lack of a linear hierarchy, and suggestive that Maslow's identified needs should be considered in a less hierarchical and more holistic light, where reflexive interactions can shift the salience and interaction of the core components. Additionally, Gambrel and Cianci (2003) note that collectivist cultures do not possess “esteem” need, as individualism is not a need within the collectivist culture.

Additionally, a longitudinal study of quality of life factors across nations tested Maslow's hierarchy of needs on a national (rather than individual) scale. Hagerty (1999) noted that although not all parts of the theory were confirmed, the sequence in which the needs were filled, in a national sense, was correlated with Maslow's hierarchical model, although working to fulfill one area did not negatively affect growth in a different area (as Maslow would have predicted). Further, Tay and Diener (2011) conducted a study regarding the needs which would be associated with subjective well-being in multiple regions of the world. The study found that specifically in African countries (along with many other regions), meeting of basic needs predicted positive life evaluations. The results also note that, as predicted by Maslow's hierarchy, people desire to meet basic physiological and safety needs before other needs. Additionally, within an African context, Jonas (2016) found that fulfillment of the physiological/basic need was rewarding for a majority of respondents. Although other studies have contradicted the hierarchical nature of Maslow's model and the assumption that one need must be fulfilled to meet a higher-level need, studies acknowledge that the basic need categories could apply to some cross-cultural contexts (Tay and Diener, 2011; Hanif et al., 2013; Jonas, 2016). Maslow's hierarchy of need is an established and oft-adopted theory for considering human behavior and forms an important starting point for considering the recovery capitals necessary for successful post-genocide recovery and healing.

## Materials and Methods

As the aim of this study is to examine how key components of recovery capital—social capital, cultural capital, physical capital, and human capital—influence the healing process of Rwandan society post-genocide, a mixed method was utilized. Specifically, we adopted a QUAN+qual simultaneous triangulation design (Morse, 1991) where surveys were collected and analyzed while supplemented with in-depth structured interviews. Thus, the results provide both quantitative and qualitative analysis of the above components of recovery capital. Below is a detailed description of the methods and measures used for this study.

### Population and Sample

In order to achieve the above aim, the study identified men and women convicted of the crime of genocide in Rwanda. Accordingly, participants in this study are convicted and incarcerated genocide perpetrators. There are approximately 30,000 individuals currently incarcerated in Rwanda for the crime of genocide (Miller, 2016), in 13 prisons. At the time of the study there were 14 prisons. One of the study site prisons (Gasabo) has closed and the prisoners relocated. Individuals convicted of the crime of genocide are housed with non-genocide prisoners. Seeking to capture geographic diversity and representativeness in the sample, three (3) prisons were selected: Gasabo prison (Kigali City), Muhanga prison (Southern province), and Ngoma prison (Eastern province). These prisons were chosen because, according to Rwanda Correctional Services (RCS), they housed high numbers of genocide perpetrators. Gasabo prison (now closed) was a male only prison, Muhanga prison houses both male and female prisoners, and Ngoma houses female prisoners only. The researchers asked prison officials to identify incarcerated men and women who were sentenced for the crime of genocide. Such a request resulted in a detailed representative sampling frame of genocide perpetrators. From these a sample was drawn of sentenced genocide perpetrators for potential inclusion in the study. Although all study participants were convicted of the crime of genocide, no data was gathered concerning specific genocidal involvement. This was because of IRB restrictions, and to avoid potential activation and distress to both participants and data collectors. Prison officials confirmed that all study participants had killed during the genocide.

After the scope and purpose of the study was explained, prisoners who agreed to participate were asked to sign an informed consent form. The prisoners were informed that their participation in the study would not impact their treatment within the prison, nor the intended day of release. The form was written in Kinyarwanda (the official language of Rwanda). Overall, and to maintain balance between the facilities 302 individuals were sampled, with 102 perpetrators sampled from Gasabo prison, 99 perpetrators sampled from Muhanga prison, and 101 perpetrators sampled from Ngoma prison. Women were over-sampled because so little information exists on female perpetrators of genocide. Estimates of how many females participated in the genocide are difficult to pinpoint. Adler et al. (2007) note that 3.4% of people incarcerated for crimes perpetrated during the genocide were women, however these data were drawn from a pre-Gacaca sample. Examining Gacaca court records, Brown (2014) estimates that approximately 6% of people incarcerated after Gacaca were women, although acknowledges that without clear acquittal data this figure remains speculative.

Not all genocide perpetrators in the three selected prisons had an equal chance of being selected into the sample. The Rwandan Correctional Service generated lists of genocide perpetrators and individuals were grouped together and asked if they wanted to participate in the study. The researchers have no data concerning whether people initially refused to participate in the study. Certainly, genocide perpetrators too sick or considered too mentally ill to participate may have been excluded by the correctional officers. Others may have been on a work group outside of the prison during the research days, and so were also not able to participate in the study.

The study was conducted in Rwanda, in 2016, and data was collected over a period of 9 days. Before the commencement of data collection, a detailed study protocol was submitted and approved by the Institutional Review Board of the corresponding authors. The researchers also received written permission from the then Commissioner General of RCS, Major General Paul Rwarakabije. A team of Rwandan data collectors received orientation to the project, were trained in the data collection protocols, and helped gather the data from incarcerated genocide perpetrators. These data collectors were members of Rwanda Center for Council Foundation, an organization committed to restorative justice practices including council process, known as “peace circles” in Rwanda. The data collectors from Rwanda Center for Council Foundation were joined by staff from ARCT-Ruhuka (Association of Rwanda Trauma Counselors), who both served as data collectors, and were available to respond in the event of distress among participants during the data gathering process.

### Data Collection

Three hundred and two perpetrators completed survey packs. It took approximately 30–40 min for participants to complete the survey pack. Survey packs included a basic demographic information form, the Kellner's Symptom Questionnaire (Kellner, 1987), and the PTSD Checklist—Civilian Version (Weathers et al., 1991). Perpetrators who were able to read and write completed the survey packs themselves during the group process. Those who experienced literacy difficulties were assisted by a Kinyarwandan-speaking data collector. Approximately 20% of participants requested help completing forms. Although some participants advised the data collectors that they had learned to read and write during their prison sentence, the illiteracy levels of the prisoners were considerably lower than the estimated 34% illiteracy rate among Rwandans at the time of the genocide (Des Forges, 1999). Specifically, data was collected on wellbeing, depression, anxiety, somatic symptoms, anger and hostility, and posttraumatic stress symptoms, as well as on standard demographics. Each of the measures are described in detail below.

### Demographic Information Form

Data collected through the demographic information form included gender, age, the district the perpetrator lived in prior to incarceration, the intended district of return, prior employment, and employment plans on release. Additionally, questions were asked concerning contact with family and friends during the prison sentence and support networks upon release. The family support data we collected are particularly relevant for examining the social capital component of recovery capital.

### Kellner's Symptom Questionnaire

Kellner's Symptom Questionnaire (Kellner, 1987) is a 92-item survey, examining both the presence or absence of symptoms and protective factors (well-being) experienced by participants in the previous 2 weeks. Items are answered in either “yes” or “no” format. The questionnaire measures the presence or absence of symptoms related to the following mental and physical health domains: depression, anxiety, anger-hostility, and somatic symptoms. Each domain is formed by 17 individual symptoms. For example, the domain anxiety comprises the following symptoms: nervous, tense/ tensed up, frightened, shaky, restless, afraid, scared, worried, terrified, takes a long time to fall asleep, jumpy, highly strung, cannot relax, panicky, frightening thoughts, feeling that something will happen, and wound up/tight. In scoring the Kellner Symptom Questionnaire one point is scored each time a participant responds “yes” or “true” to an item. The Kellner Symptom Questionnaire also examines aspects of wellbeing, specifically contentment, relaxation, friendliness, and somatic well-being. One point is scored each time a participant responds “no” or “false” to one of the items on the well-being subscale.

The Kellner Symptom Questionnaire was translated into Kinyarwanda by the data collection team and was validated for its accuracy separately by a minimum of three speakers fluent in both English and Kinyarwanda. The survey was then pilot-tested with a small sample of incarcerated genocide perpetrators in Gasabo prison in 2015. Adjustments were then made to the final version of the translated Kellner's symptom questionnaire.

Research indicates that the Kellner's Symptom Questionnaire has high reliability across the depression, anxiety, anger-hostility, and somatization domains (Teicher et al., 2015). The depression subscale is characterized by a Cronbach's Alpha = 0.94, anxiety = 0.92, anger-hostility = 0.91, and somatic symptoms = 0.86 (Kellner, 1987; Rafanelli et al., 2000; Thompson et al., 2004).

### Posttraumatic Stress Disorder Checklist-Civilian Version (PCL-C)

The civilian version Posttraumatic Stress Disorder checklist (PCL-C) (Weathers et al., 1991; Weathers, 2008) is a widely used, standard self-report measure of posttraumatic stress disorder (PTSD). The items measured correspond with PTSD diagnostic criteria outlined in DSM-IV. The PCL-C measurement is a self-report rating scale—ranging from (1) “not at all” to (5) “Extremely.” Participants assess the degree to which they have been bothered by particular problems related to “past stressful experience” in the previous month. For Rwandan genocide perpetrators we adapted the PCL-C changing the words “a stressful experience from the past” with “the genocide,” where necessary. Statements rated therefore included “Suddenly acting or feeling as if the genocide were happening again (as if you were reliving it)” and “Avoiding activities or situations because they reminded you of the genocide.” The same translation process adopted for the Kellner Symptom Questionnaire was used to translate the PCL-C into Kinyarwandan. Previous studies found that the overall reliability of the PCL-C is very high (Cronbach's Alpha = 0.96) (Conybeare et al., 2012; Skeffington et al., 2016).

Levels of genocide-related posttraumatic stress, indicated by the PCL-C scores of participants, were used as a proxy for the concept “post-genocide healing” in this study. Schaal et al. (2012) suggest that perpetrators who continue to relive their genocidal actions and experiences more than 20 years after the genocide, through nightmares, flashbacks and thought preoccupation were more likely to desire peaceful coexistence with former enemies than those with lower levels of posttraumatic stress. Certainly, the PCL-C scores could be used as an independent variable in this study—forming a component of human capital—however, given that physical and mental health symptoms provided through the Kellner Symptom checklist were extremely thorough, we felt this element of physical and mental health was not lacking. Furthermore, the PTSD symptoms measured through the PCL-C differ from the physical and mental health symptoms measured thorough the Kellner in that they are symptoms directly related to the genocide. For example, the PCL-C examines the frequency of physical reactions (e.g., heart pounding, trouble breathing, or sweating) of the perpetrator when reminded of the genocide during the previous month, whereas the Kellner examines whether they have experienced “heart beating fast” or “breathing difficult” during the previous 2 weeks.

A choice was made to focus only on incarcerated genocide perpetrators, because of limited funding and access, and an expressed desire from Rwanda Correctional Services to better understand the needs of incarcerated perpetrators. Few studies have examined the experiences of incarcerated perpetrators, and in-relation to post-genocide healing such research seeks to develop understandings which transcend limiting victim-perpetrator dichotomies.

### Qualitative Structured Interviews

As mentioned earlier, and in order to gain better insight into the experiences of those sentenced for their part in the genocide, 25 structured interviews were conducted in each prison—Muhanga, Ngoma, and Gasabo—totaling 75 interviews in all. Specifically, 50 males and 25 females participated in the in-depth structured interviews. At the time of the interview, they had served an average of 14^3/4^ years in prison for their participation in the genocide. The interviewee sample was drawn randomly from the original sample of 302 génocidaires. The consent process was explained to each of the participants to be interviewed, and a new signed consent form was acquired for each in-depth structured interview participant. Participants were interviewed by two data collectors, with one asking questions from an interview protocol in Kinyarwanda, and the other, fluent in both Kinyarwanda and English recording responses in English. Each interview lasted approximately 30 min.

Overall questions examined readiness for release from prison, and as such focused on the various aspects of social capital, cultural capital, physical capital, and human capital as means to gain better understanding of recovery capital. More specifically, interviewees were asked to share the ways in which they manage feelings associated with their traumatic experiences as genocide perpetrators, and their perception of available support upon release. Examples for some open-ended questions that reflect the above are: Have you received any support or attended any programs during your time in prison? Imagine your day of release, what do you think you will do? How do you think those on the outside are going to react to your release? What are your biggest concerns about release? Tell me about your support network. Who will be there for you when you are released? Each question had subsequent prompts to help respondents focus on relevant information.

### Constructing Recovery Capital and Post-genocide Healing

Both surveys and interviews affirmed the construct of key variables of recovery capital aimed to examine the relationship between social, cultural, physical, and human capital and post-genocide healing (see Table 2). Social capital is captured through the prison visitation questions in the demographic information form and interview questions relating to programs received in prison and networks of support for impending release. Cultural capital is explored through responses to interview questions concerning perceptions of how the genocide perpetrators will be received by the community when they are released from prison. The questions are a tentative step toward capturing the “values, beliefs, dispositions, perceptions, and appreciations” (Cloud and Granfield, 2008, p. 1974) of perpetrators of genocide as they draw on diminished stocks of cultural capital while they navigate community reentry. The variable human capital is drawn from responses to the Kellner Symptom Checklist, focusing on the physical and mental health symptoms and the protective factors of the prisoners. Physical capital is captured in interview responses to specific identified needs related to successful community reintegration. Finally, insight into our dependent variable is garnered from responses to the PCL-C checklist. Quantitative measures were chosen for social and human capital and qualitative measures were chosen for cultural and physical capital. This was because the components of cultural and physical capital were focused on perceptions of the post-release future rather than current experiences, and therefore were better captured through the interview method adopted.

**Table 2:** Measuring components of recovery capital.

**Type of capital**	**Study measures**	**Method**
Social capital	Frequency of visits from family or friends Perceived networks of support	Survey Interview
Cultural capital	Perceived ease of reintegration post-release	Interview
Human capital	Kellner Symptom Checklist	Survey
Physical capital	Perceived needs for successful reintegration	Interview

The quantitative data was entered into spreadsheets and analyzed using SPSS version 23 (SPSS, 2016). Specifically, binominal correlation coefficients were calculated to examine the relationship between components of social capital (prison visitation), human capital (physical and mental health symptoms) and levels of genocide-related posttraumatic stress. Linear regression models were then run to examine the relationship between social and human capital variables and posttraumatic stress. Interview responses were typed into an excel spreadsheet. The responses were line-by-line coded by the first author and two trained undergraduate students. The coders varied in terms of age, gender, ethnicity, and background experience. The initial level codes were then examined using the “constant comparison” method (Glaser and Strauss, 1967), to determine whether the codes assigned reflect the same concept. Effort was made to capture the originality within the data by ensuring that the codes were generated by the interview responses rather than the data being simply mined for themes compatible with a pre-existing theoretical lens (Rubin and Rubin, 2005).

## Results

### Descriptive Data

Of the 302 incarcerated genocide perpetrators sampled for this study 180 perpetrators were male (59.6%) and 122 were female (40.4%). More than half of our sample (54.6%) were between the ages of 45–59 years old, and just over one-third (33.7%) of the sample were older than 60. The age data indicates that the majority of perpetrators in the sample were between 23 and 40 years of age at the time of 1994 Rwanda genocide.

All of the genocide perpetrators in the sample were sentenced. Sentence lengths were calculated by asking what year the perpetrator entered the prison, and what year he or she will be released. Just under a quarter of the sample were unable to provide a potential release date. The overall mean sentence length in years for those who had a release date in the sample was *M* = 16.47 (*SD* = 4.56, range = 3–30). There were no statistically significant differences in length of sentence between males and females. The majority of perpetrators in the sample (87.4%) were farmers prior to their incarceration. Others were teachers, chefs, nurses, bakers, and bus attendants. The majority of perpetrators (83.1%) intended to become subsistence farmers when they leave prison.

### Social Capital of Genocide Perpetrators

More than two-thirds of our sample of perpetrators (64.6%) reported receiving regular visits from family or friends during their sentence. Family visitation on Fridays is encouraged by the Rwanda Correctional Service, and facilitates prisoners' access to special diets and medicine. We asked perpetrators about their perception of the frequency of family and friend visits. Surprisingly, 55% of the sample felt that they had been “hardly visited” or “never been visited.” The apparent discrepancy between the reports of receiving “regular” visits and receiving few or no visits may be due to translation issues regarding the words “visit” and “contact.” Alternatively, it suggests a disconnect between a desire for connections with family and friends and the reality of prison visitation. The relationship between the degree of support while incarcerated and levels of current posttraumatic stress was not statistically significant.

Interviews regarding social supports indicated that the majority of perpetrators had some level of bonding social capital, naming spouses, children, or siblings as their primary support network when released. As one perpetrator describes: “My big family will help me to build myself. And I think they will help me reintegrate quickly in their daily life” (ID: 3006; male). Although the bonds were present, many perpetrators qualified the bond by recognizing the challenges their support network was experiencing. A husband was identified as one perpetrator's social support, “…but he is too old and sick” (ID: 2061; female), a wife was named as the central figure in one perpetrators' network “…but she is also infected with HIV” (ID: 1006; male), a child could help “…but he is poor also” (ID: 2077; female). Overwhelmingly, the efficacy of bonding social capital was challenged by family and friends being sick, poor, and/or old. Other perpetrators were unsure whether their family was willing to support them: “I have a big family, some are businessmen and I have one other brother in the military, but I am not 100% sure of their support.” (ID: 1084; male). Concerns about family members who had divided and sold their land or formed new relationships and had additional children during their period of incarceration, contributed to the uncertainty of pre-existing bonding social capital. Others acknowledged that they had no identified social supports and thus were relying on government officials to assist on their release. One perpetrator hopes for linking social capital: “The one to support me is the Rwandan government. I have nothing like any support or network.” (ID: 2054; female).

Given the uncertainty regarding bonds with family and friends, it is perhaps unsurprising that the majority of perpetrators cited the relationships they had formed with other prisoners as critical social capital they bring to the reentry process. One perpetrator describes the importance of maintaining relationships with his fellow prisoners: “I met different people here. Some became friends, we shared almost everything. So, I don't think when I am released, I will hate. I will stay connected with them.” (ID: 1089; male). The perpetrators spoke of other prisoners teaching them to read, write, and weave baskets during their incarceration. One prisoner acknowledges that the support he has received from other prisoners will help him when he is eventually released: “Other prisoners taught me how to read and write. They taught me how to behave on different challenges that may come as I go home.” (ID: 1015; male). The majority of prisoners had plans to continue visiting their friends in prison after they were released. As one perpetrator explains: “The other prisoners we pray together, we are friends, I will stay connected to them because they have become a part of who I am.” (ID: 1004; male).

### Cultural Capital of Genocide Perpetrators

Examining responses to interview questions about how the community may respond to their release provides insight into the degree of cultural capital held by the genocide perpetrators in the sample. Many génocidaires aspired to work alongside fellow Rwandans to help rebuild their country, and develop themselves and their family. Some hoped to find family members whom they had lost contact with, while others aspired to join farming cooperatives. Most of those interviewed did not fear retaliation from community members, as they reported that their apology and requests for forgiveness had been accepted by the victim's families. As one genocide perpetrator states: “People from outside will have no negative reaction to me because I asked for forgiveness to all and now many of them visit me at prison. I don't have any worry regarding my release because I even wrote a letter to my family and all neighbors requesting to be forgiven.” (ID: 2007; female). For another, although forgiveness was already received, further demonstrations were considered necessary: “I don't think there is anything bad regarding people from outside because I asked to be forgiven and they forgave me. The people who made me imprisoned were my family because it was my brother that reported me. If I find my victim's wife has no child I can give her mine so that he can help her.” (ID: 2050; female). Other perpetrators were scared about not being welcomed back by neighbors because they had yet to connect with the victim's family, and ask for forgiveness. One perpetrator explains: “I used to live in peace with my neighbors, so I have no doubt that they will welcome me, what I am looking for is to go out and find the people I betrayed and ask for forgiveness” (ID: 2061; female).

### Physical Capital of Genocide Perpetrators

The need for housing when released was one of the biggest concerns identified by the genocide perpetrators during interview. Former housing was destroyed either during the genocide, or fallen into disrepair in the ensuing decades. One perpetrator explains: “My house was destroyed. I will need to see how I can get another shelter” (ID: 3033; male). Another laments: “I need also shelter because my house was destroyed” (ID: 2100; female), while another perpetrator explains: “I am worried about poverty because everything was destructed, including my house and my animals were stolen” (ID: 2046; female). Money to help meet immediate physiological needs such as shelter and food were a big concern, as well as money to purchase land, animals, and tools. Some of the genocide perpetrators considered themselves fairly rich in physical capital, and therefore felt assured that their reintegration would be relatively smooth: “I have got a forest so it will help me a lot. My land is big enough once I do modern farming it will be very helpful” (ID: 2057; female). For a small number of perpetrators thinking about their future was overwhelming: “I am so much concerned about where I will start from, I came to prison when I was very young, my agemates have developed. It is as if my future was cut down. I have so many plans but I don't know where to start. I don't have [a] starting point” (ID: 3027; male).

### Human Capital of Genocide Perpetrators

The Kellner Symptom Questionnaire provides considerable information concerning the mental and physical wellbeing of the genocide perpetrators. In relation to depression, 39.6% of the sample reported eight or more depressive symptoms and 10.2% of our perpetrators sample reported scores that are higher than 12. Depression scores ≥ 12 are considered clinically significant (Teicher et al., 2010). Approximately one-third of our sample (32.2%) had abnormally high anxiety scores. Overall the sample had extremely low scores in the anger-hostility domain, with the mean score being 1.57 out of a possible 17. The perpetrators raised levels of somatic symptoms. Just under two-thirds of the sample (62.8%) reported that their body had felt numb or tingling, and a little over a half (53.2%) had felt nauseated. Just under half had experienced cramps (42.9%), upset bowels, (36.0%) or were having difficulty breathing (38.4%).

The genocide perpetrators had elevated PTSD symptom scores, ranging from 16 to 79 out of a possible 85 (*M* = 40.51, *SD* = 12.86). Cut-off scores of 30–35 are suggested for civilian populations as indicators for PTSD. Typically, the prevalence of PTSD among the general population in the U.S. is <15%, with prevalence in Veterans Administration primary care and specialized medical clinics between 16 and 39% and in VA or civilian mental health clinics above 40% (National Center for PTSD, 2012). In our sample, more than 66.6% of genocide perpetrators scored above 35. Some 22.8% of the sample had a score higher than 50. Experience of PTSD symptoms was highly and significantly correlated with our measures of human capital (mental health and somatic symptoms). Increases in reported anxiety and depression symptoms were associated with increases in posttraumatic stress symptoms. Interestingly, lower somatic symptom scores were significantly associated with higher levels of posttraumatic stress symptoms. Perpetrators with fewer somatic symptoms experienced higher levels of posttraumatic stress. There was no statistically significant relationship between a perpetrators degree of social capital, measured by frequency of visitation, and level of posttraumatic stress symptoms.

### Findings From the Linear Model Examining the Effects of Social and Human Capital on Healing

Using quantitative data acquired from the surveys, human capital was constructed by examining depression, anxiety, and somatic symptoms experienced by the participants in the previous 2 weeks. These were drawn from answers to the Kellner Symptom Questionnaire, as discussed previously in this paper. Social capital was measured by the reported frequency of contacts from family and friends during incarceration, drawn from the demographic information form. Answers to the question: “How often have you had contact with a family member or friend while you have been in prison?” were originally measured using a four-point Likert-type scale, ranging from “Never,” “Hardly at all,” “Quite a bit,” and “A lot.” Given the spread of responses across the categories, responses were dichotomized into low social capital (those who responded “Never or hardly at all”) and high social capital (those who responded “Quite a bit or a lot”), to ensure better weighted categories. Our quantitative measurement for post-genocide healing was the level of posttraumatic stress symptoms experienced by the perpetrator in the previous month. This data was captured in the PCL-C questionnaire.

We examined correlations between our measures of human and social capital and PCL-C scores. Positive, strong and significant correlations were found between PCL-C scores and our key components of human capital: depression (*R*_*Pearson*_ = 0.588, *p* ≤ 0.001), anxiety (*R*_*Pearson*_ = 0.581, *p* ≤ 0.001), and somatic symptoms (*R*_*Pearson*_ = 0.389, *p* ≤ 0.001). A non-significant negative correlation was found between PCL-C scores and our measure of social capital.

In order to examine the effects of human and social capital as our independent quantitative variables and posttraumatic stress symptoms, as the study dependent variable, two separate linear regression models were calculated. Specifically, linear regressions models were calculated for those with high levels of social capital (e.g., response of “Quite a bit or a lot” to the question on the frequency of visits while incarcerated), and low levels of social support (e.g., response of “Never or hardly at all” to the question on the frequency of contact while incarcerated). Both models were found to be statistically significant [*r*^2^ = 0.423, (adjusted *r*^2^ = 0.406), *F*_(3, 107)_ = 26.110, *p* < 0.001, and *r*^2^ = 0.375, (adjusted *r*^2^ = 0.364), *F*_(3, 143)_ = 28.577, *p* < 0.001 respectively]. Examining Table 3, the models demonstrate that the components of human and social capital are significantly related to healing after genocide, although the direction of the relationship varies with high and low social capital. Specifically, examining the model for high social capital reveals a better ability to explain the variation in the dependent variable (e.g., healing/ recovery) compared to the model for low social capital, as can be seen by the adjusted *R*^2^. A careful examination of the effects of the constructs of human capital reveal that depression and anxiety are both statistically significant factors that positively contribute to posttraumatic stress. Reducing depression and anxiety is likely to aid post-genocide healing and therefore the healing process. In other words, higher levels of human capital are positively connected to healing and recovery. While not statistically significant, the model provides a glimpse into the negative effect of physical illness on healing and recovery from trauma.

**Table 3:** General Linear Models examining the association between human capital and posttraumatic stress symptoms by the level of social capital.

	**Low social capital (*****n*** **=** **166)**			**High social capital (*****n*** **=** **134)**		
**Human capital variables**	**B (SE)**	**Beta**	***t***	**B (SE)**	**Beta**	***t***
Depression	0.969 (0.388)	0.278^**^	2.500	1.369 (0.400)	0.384^***^	3.427
Anxiety	1.016 (0.324)	0.336^**^	3.131	1.312 (0.353)	0.409^***^	3.721
Somatization	0.138 (0.244)	0.049	0.564	−0.414 (0.268)	−0.148	−1.544
Adjusted *R^2^*	0.362 (10.142)^***^			0.406 (10.058)^***^		

** p < 0.010

*** *p < 0.001*.

## Discussion

The concept of recovery capital, comprising of social, cultural, human, and physical capital is a useful, nuanced construct for considering the conditions necessary for the peaceful reintegration of Rwandan genocide perpetrators after prison. The genocide engendered a perversion of social capital, whereby bonding social ties were strengthened through groups dedicated to killing, torturing, and looting. Inter-group bonds were strengthened by the dissemination of genocidal ideology and hatred. Simultaneously, bridging ties particularly with diverse groups were severed. In our sample the genocide perpetrators were relying on existing social ties to assist them with their impending community reintegration, however they were cognizant their involvement in the genocide and subsequent conviction and sentencing strained previously strong social ties. Unsurprisingly, given the length of sentence for many of the perpetrators, friendships nurtured in prison formed a significant proportion of perceived social capital on release. Such bonding social ties could be considered to be akin to traditional substance abuse recovery relationships, where those struggling with addiction form supportive relationships with those experiencing similar problems. Perpetrators identified a need for vertical linking ties with government officials to assist with their reintegration process. Certainly, efforts made by local cell and sector leaders to genocide perpetrators in prison is an important step toward expanding the social capital available to prisoners.

We have observed that just as there is a perversion of social capital during genocide, cultural capital is similarly perverted. Genocidal ideology based on fear and hatred of the other is transformed and concretized through the same mechanisms through which cultural capital is transmitted. The Rwandan government has made considerable efforts to facilitate a shared cultural narrative of unity and reconciliation since the genocide. The perpetrators in our sample described receiving anti-genocide ideology programming during their incarceration, including the “I am Rwandan” program, the “Unity and Reconciliation” program, patriotism programming, the conflict resolution classes. In addition, many perpetrators discussed listening to the national radio soap-opera “Musekewaya,” with its messaging of acceptance, inclusion and active bystandership against group violence. Such steps help to strengthen cultural capital to minimize the growth of genocidal ideology.

Genocide smashes physical capital, destroying basic infrastructure and depriving people of livelihoods. For many genocide perpetrators approaching release from prison there was considerable uncertainty concerning how they would achieve basic needs of food, shelter, and security. Those achieving some level of security spoke of wanting to build networks and ties, and live harmoniously with others.

For survivors of genocide, whether perpetrators or victims, human capital is significantly impacted. There was a high prevalence of mental health symptoms in our sample, with raised levels of anxiety and depression. Posttraumatic stress symptoms related to participation in the genocide were both widespread and severe. Unhealed psychological wounds may promulgate genocidal ideology and make unity and reconciliation unlikely (Staub, 2015). The high numbers of somatic symptoms experienced by the prisoners could certainly be the result of overcrowded prison conditions and limited nutrition and health care, however the common symptoms described including numbness and tingling, nausea, upset bowels, difficulty breathing and heavy arms and legs are symptoms often associated with stress, distress, and trauma. Indeed, it could be tentatively argued that our findings from the linear regression models support these findings as somatization was negatively related to high level of social support and thus perhaps negatively affecting the healing process. Although not statistically significant, perhaps owing to the small sample size, it is reasonable to consider that the gains achieved by social support on post-genocide recovery are negatively impacted by high levels of physical health problems.

It is clear that in relation to Maslow's hierarchy of needs, the first three levels: physiological needs, safety needs, and esteem are critical for the development of post-genocide recovery capital among genocide perpetrators. The incarcerated perpetrators require nurturing forms of social, human, and physical capital to successfully reintegrate into society after their prison sentences. Social ties, with both existing family members and bonds developed during their years of post-genocide incarceration were identified as a critical re-entry need. The higher levels of Maslow's hierarchy, self-actualization and self-transcendence, are threatened by genocide-influenced perversions in social and cultural capital. Whether such perversions can be neutralized in post-genocide Rwanda through a top-down unity and reconciliation narrative remains moot. Certainly, efforts to build individual social ties between perpetrators and survivors are important steps toward ensuring that all Rwandans can realize and fulfill their potential.

Although recovery capital is a useful concept for considering post-genocide trauma recovery, it may be more pertinent for future researchers to consider whether a resilience model rather than a recovery model best captures the interconnectivity of social, cultural, human, and physical capital after trauma. Through a recovery lens the components of recovery capital become stocks from which to draw. With enough investments the individual can heal, and to be healed is a return to the pre-trauma or pre-addiction state. The concept of resilience capital perhaps provides a richer, more dynamic, and certainly more generative appreciation of how individuals not only survive traumatic genocidal experiences, but also make positive meaning going forward.

There are several limitations to this study. First, although efforts were made to draw our sample from different geographic locations in Rwanda, sampling error may have occurred. Generation of a complete sampling frame of all genocide perpetrators was neither practical or feasible. Secondly, the measures used were developed and validated in western contexts. Although the authors involved Rwandan partners extensively in the translation (and hence initial interpretation), and validation of the instruments, groups of symptoms may not be experienced as specific constructs (anxiety, depression and so forth) within the Rwandan context. In particular, physical symptoms of posttraumatic stress may vary in different cultural contexts (Rasmussen et al., 2007). We did, however, ensure that the tools used were locally agreed upon and culturally calibrated. Another limitation is our use of levels of PTSD symptoms as a proxy for post-genocide healing. Posttraumatic stress levels could certainly have been used as an independent variable rather than the dependent variable in this study, as posttraumatic symptoms could be considered an element of human capital.

A considerable strength of the study is the mixed methods design involving the collection of survey data from 302 incarcerated genocide perpetrators in Rwanda, triangulated with in-depth structured interviews with 50 male and 25 female perpetrators. Together the data provide a nuanced understanding of the challenges faced by individuals as a country moves beyond genocidal ideology. Recovery after genocide requires the strengthening of social, cultural, physical, and human capital, a daunting endeavor when important social assets can be perverted or destroyed during the violence. Bringing the lens of recovery capital to our examination of post-genocide recovery can accentuate resiliency, highlight deficits and strengths in the recovery process, and identify critical areas where services are needed.

## Ethics Statement

This study was carried out in accordance with the recommendations of CUNY's Human Research Protection Program. The protocol was approved by the John Jay College of Criminal Justice's Institutional Review Board (protocol #: 2015-0825). Partial funding for the study was provided by a Seed grant from the Office for the Advancement of Research at John Jay College. All subjects gave written informed consent in accordance with the Declaration of Helsinki. The specific consent procedure was as follows: Participants were taken to a hall within the prison and the study procedures were explained in the local language (Kinyarwanda). Translated consent forms were given to participants. The consent form was read by a local representative from Rwanda Center for Council. Participants willing to participate in the study signed the consent form.

## Author Contributions

KB-C contributed to the conceptualization and design of the study, collecting the data in Rwanda, statistical analyses, interpretation of the data, drafting of the article, and review and revision of the article. LG contributed to the statistical analyses, interpretation of data and discussion section. LL contributed to the conceptualization and design of the study, pilot-tested the measures in Rwanda, interpreted the data, and critically reviewed and revised the article. KY contributed to the literature review, and the critically reviewed and revised the article. All authors approved the final article as submitted.

### Conflict of Interest Statement

LL is the Director and co-founder of Threshold Global Works (TGW). The mission of Threshold GlobalWorks is to accelerate leading-edge initiatives, informed by neuroscience, that amplify social resilience and human potential. LL does not have any conflict of interest with this paper and there is no foreseeable monetary profit from publication of this paper. The remaining authors declare that the research was conducted in the absence of any commercial or financial relationships that could be construed as a potential conflict of interest.
